# Supplementary material for: Single cell and spatial sequencing define processes by which keratinocytes and fibroblasts amplify inflammatory responses in psoriasis
Source: Nat Commun. 2023 Jun 12;14:3455. doi: 10.1038/s41467-023-39020-4 (PMC10261041; doi:10.1038/s41467-023-39020-4)
Supplement: Supplementary file 1 — Supplementary Information [file 41467_2023_39020_MOESM1_ESM.pdf]

Supplementary Fig. 1

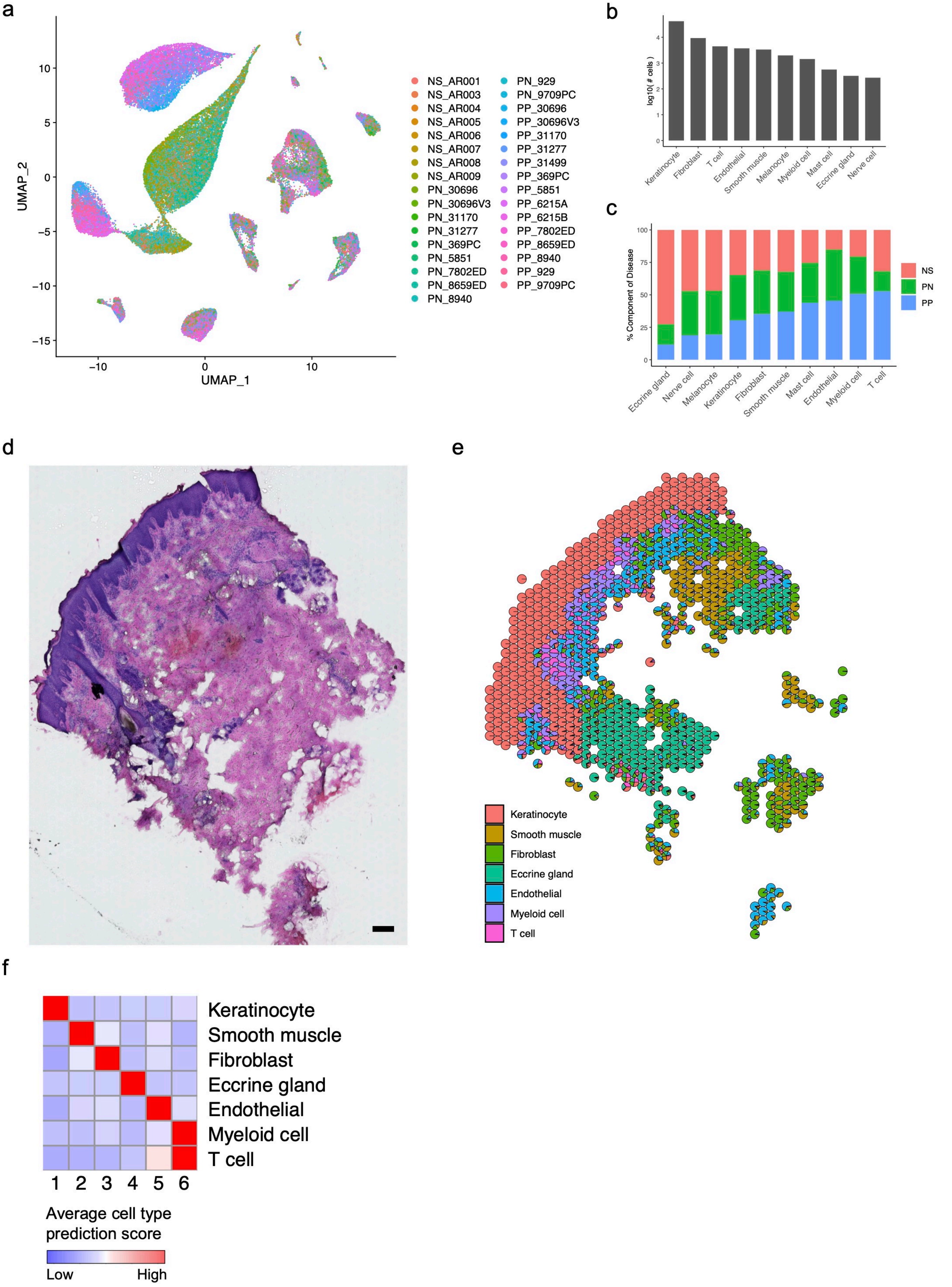

Supplementary Fig. 1. Spatial sequencing localizes the cell types in the psoriasis skin sample.

a. UMAP plot showing the cells colored by sequencing libraries.

b. Bar plot showing the log10 number of cells for each cell type in scRNA-seq.

c. Bar plot showing the abundance composition across the disease conditions for each cell type in scRNA-seq.

d. H & E staining of the PP biopsy used for spatial sequencing.

e. Scatter pie plot showing the cell type composition of the PP spatial-seq sample. Each spot is represented as a pie chart showing the relative proportion of the cell types.

f. Heatmap showing the average cell type prediction score for each cluster.

Supplementary Fig. 2

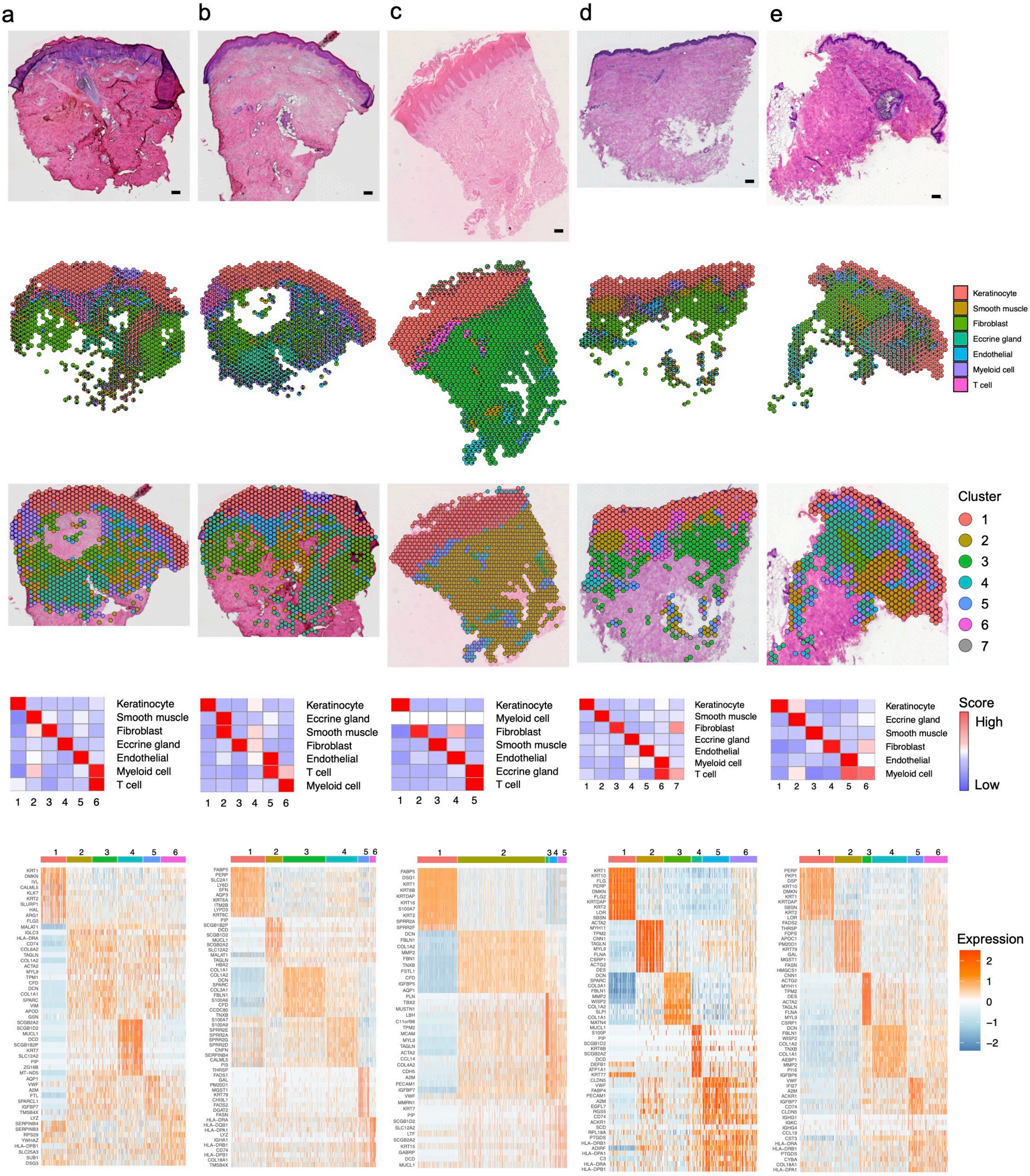

Supplementary Fig. 2. Spatial sequencing localizes the cell types in two more psoriasis and two healthy skin samples.

a. Spatial sequencing for the second PP sample (PP2). First panel: H & E staining of the biopsy used for spatial sequencing; Second panel: scatter pie plot showing the cell type composition; Third panel: clustering of the spots; Fourth panel: heatmap showing the average cell type prediction score for each cluster; Fifth panel: heatmap showing the top marker genes for each cluster, the color scale represents the scaled expression of the gene.

b. Spatial sequencing for the third PP sample (PP3). The panels follow the same arrangement as in a.

c. Spatial sequencing for the fourth PP sample (PP4). The panels follow the same arrangement as in a.

d. Spatial sequencing for the first NS sample. The panels follow the same arrangement as in a.

e. Spatial sequencing for the second NS sample. The panels follow the same arrangement as in a.

Supplementary Fig. 3

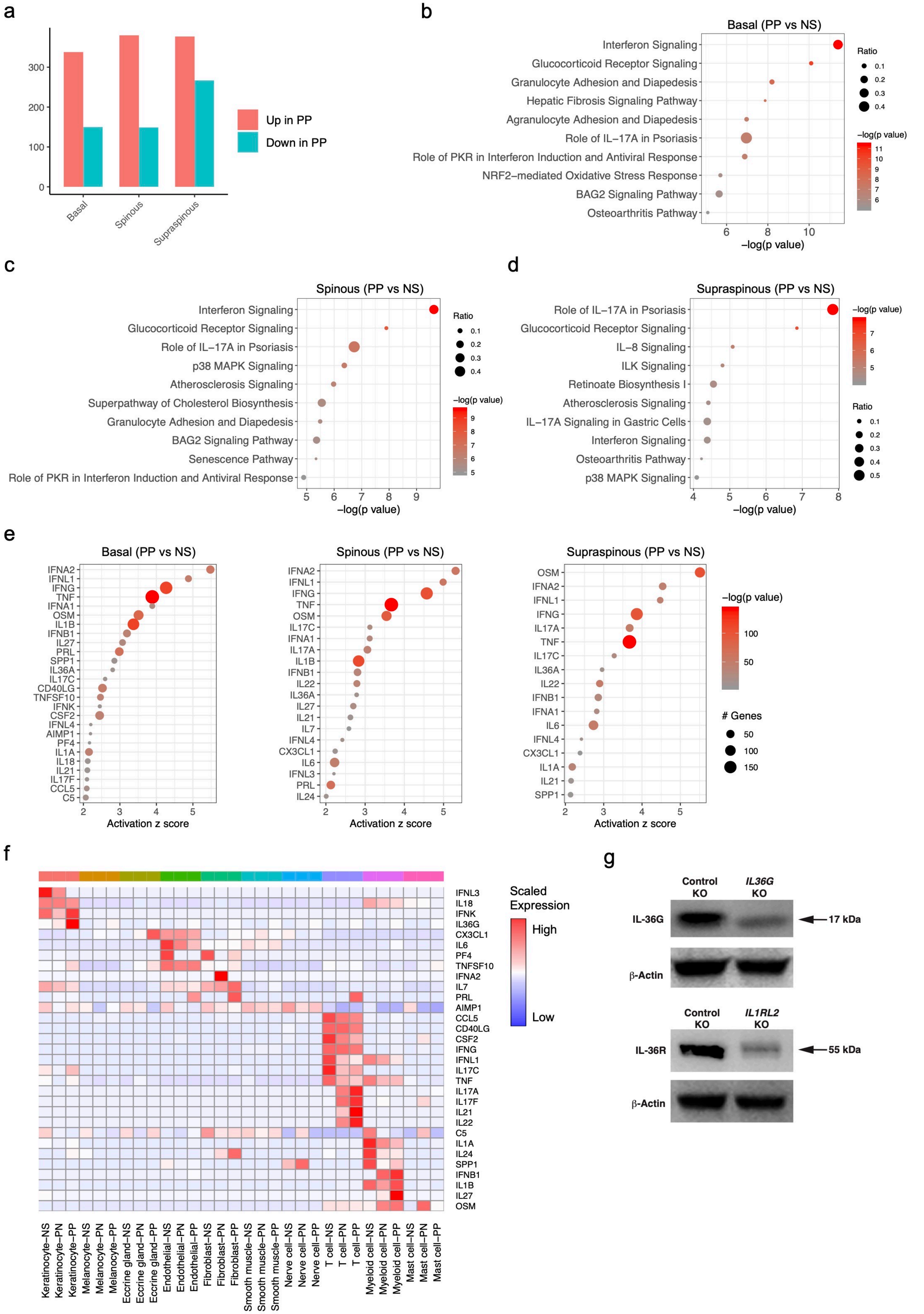

Supplementary Fig. 3. Differential expression analysis in keratinocyte subtypes reveals potential upstream regulators.

a. Bar plot showing the number of differentially expressed genes comparing PP to NS in the keratinocyte subtypes.

b. Dot plot showing the top 10 canonical pathways enriched using the differentially expressed genes (DEGs) comparing PP to NS in basal keratinocytes. The color scale represents the -log10(p value) from the enrichment analysis. The size of the dot represents the ratio calculated by dividing the number of associated genes found in the DEG list by the total number of genes in each pathway. Hypergeometric test was used for the enrichment analysis, and the Benjamini-Hochberg Procedure was used for false discovery rate adjustment. The same test and procedure were applied in c, d, and e.

c. Dot plot showing the top 10 canonical pathways enriched using the DEGs comparing PP to NS in spinous keratinocytes.

d. Dot plot showing the top 10 canonical pathways enriched using the DEGs comparing PP to NS in supraspinous keratinocytes.

e. Dot plot showing the upstream regulators for the DEGs identified in each keratinocyte subtype by comparing PP to NS cells. The color scale represents the -log10(p value) from the enrichment analysis. The size of the dot represents the number of differentially expressed genes in the downstream of the upstream regulator.

f. Heatmap showing the average expression of the upstream regulators in each cell type separated by the disease conditions. The color scale represents the scaled expression of the gene.

g. Western blot showing the knockout of *IL36G* and *IL1RL2* in keratinocytes. Source data are provided as a Source Data file.

Supplementary Fig. 4

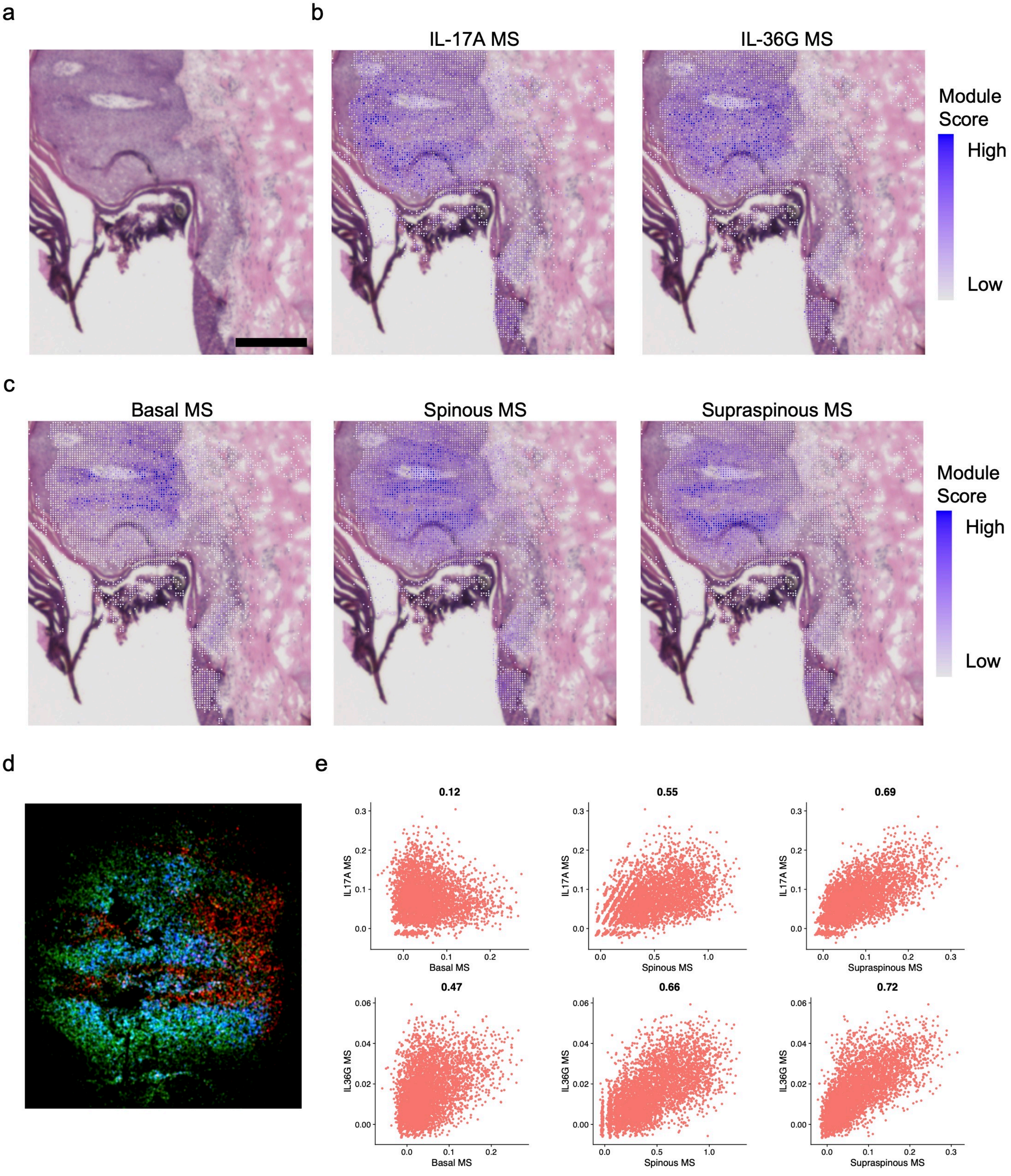

Supplementary Fig. 4. IL-17 and 1L-36 responses were strongest in the supraspinous keratinocytes in psoriasis.

a. H & E staining of the PP biopsy used for Seq-Scope profiling.

b. Module scores of IL-17A and IL-36G in the PP skin biopsy specimen processed by Seq-Scope.

c. Module scores of the keratinocyte subtypes in the PP skin biopsy specimen processed by Seq-Scope.

d. Three keratinocyte subtype scores in one plot. Red: Basal score. Blue: Spinous score. Green: Supraspinous score.

e. Scatter plot showing the correlation between the subtype module score and the IL-17A or IL-36G module score.

Supplementary Fig. 5

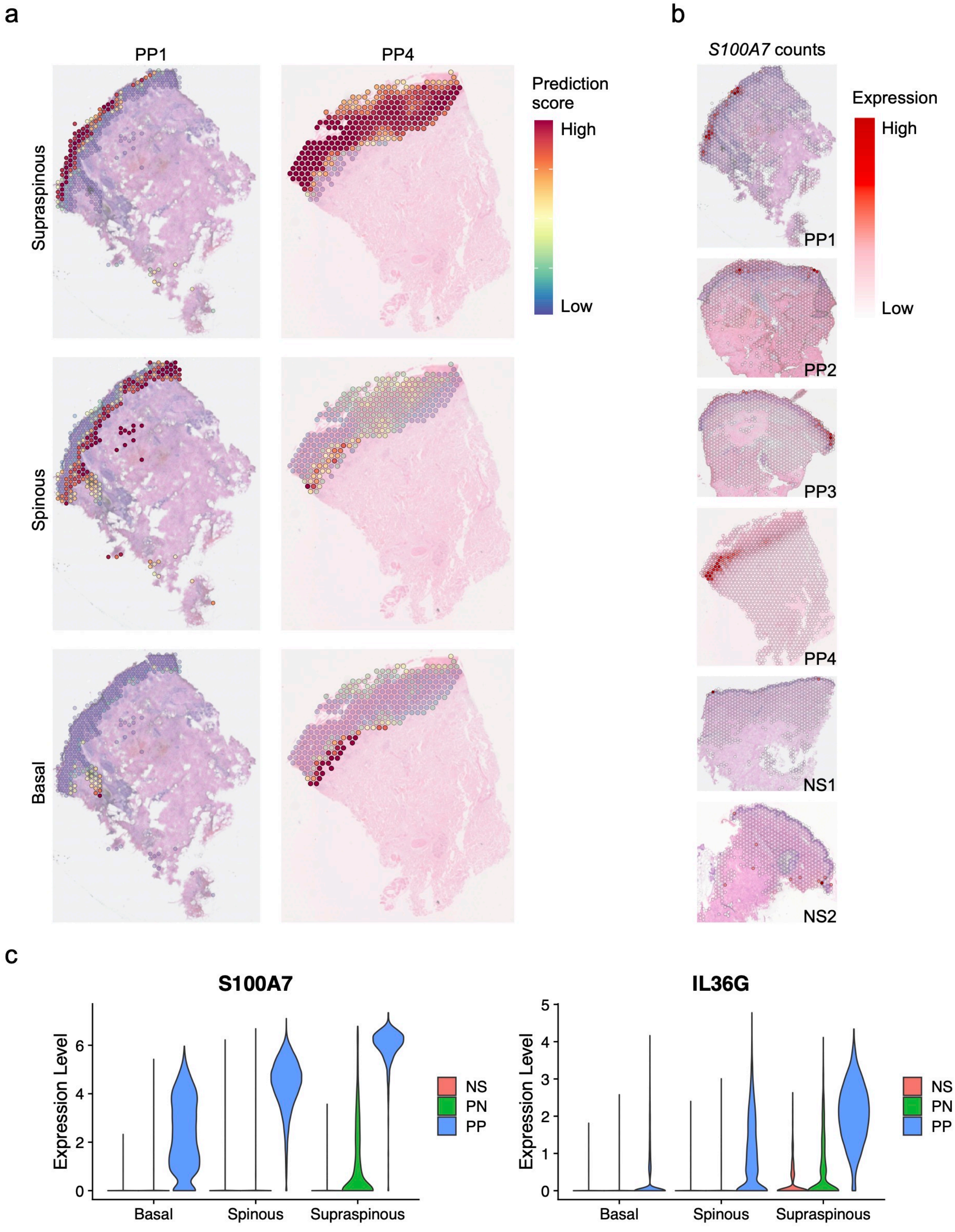

Supplementary Fig. 5. Spatial location of the IL17 and TNF responses.

a. Spatial plots showing the keratinocyte subtype prediction scores. Only the spots in the keratinocyte cluster were plotted.

b. Spatial plots showing the raw expression counts for *S100A7* in all the spatial-seq samples.

c. Violin plot showing the expression level of *S100A7* and *IL36G* in the keratinocyte subtypes in the scRNA-seq data, and each subtype is split by the disease conditions.

Supplementary Fig. 6

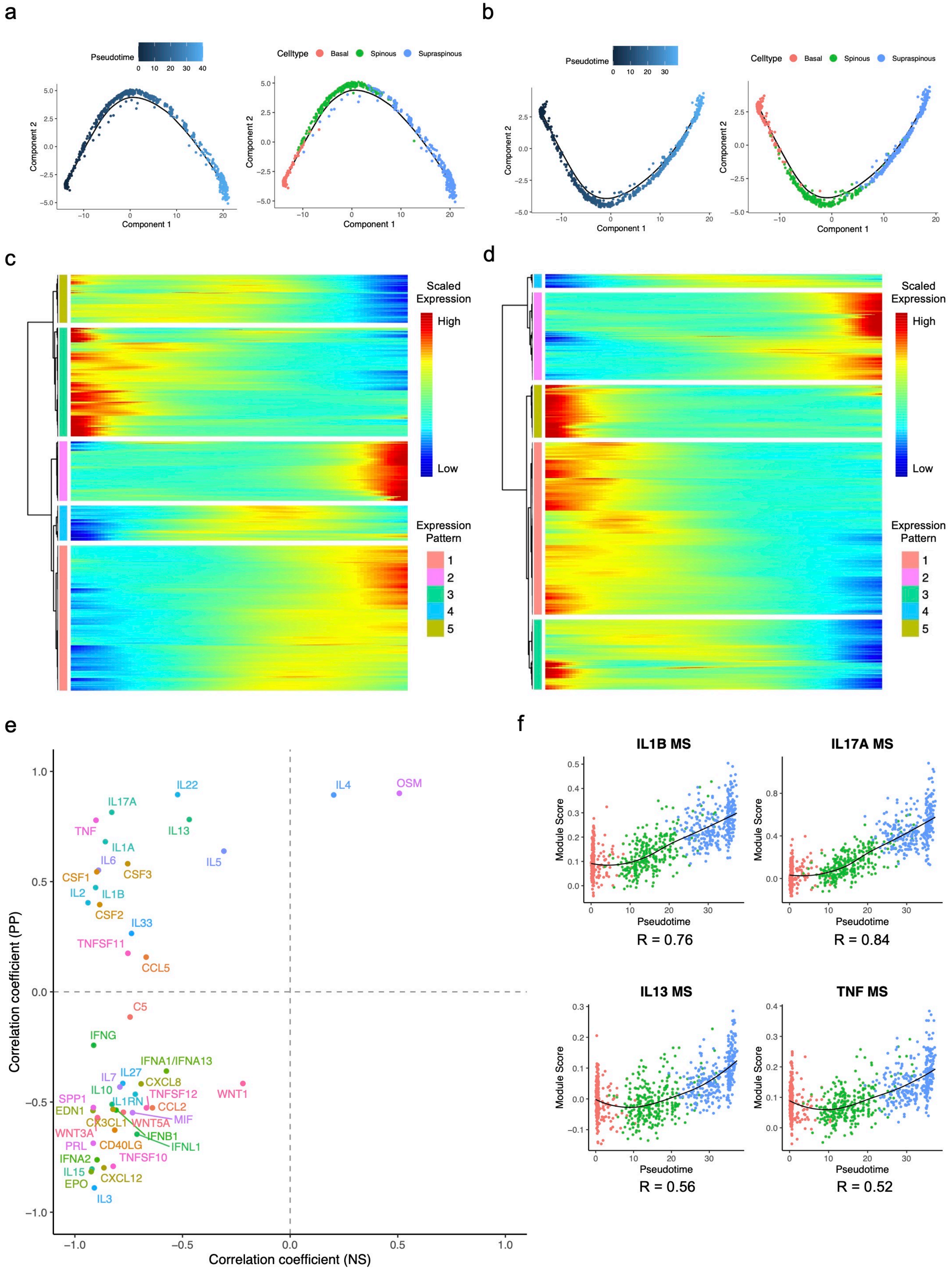

Supplementary Fig. 6. Pseudotime construction in NS and PP keratinocytes.

a. Pseudotime trajectory colored by the pseudotime (left) or subtype identity (right) of the NS keratinocytes.

b. Pseudotime trajectory colored by the pseudotime (left) or subtype identity (right) of the PP keratinocytes.

c. Heatmap showing the five expression patterns of variable genes along the pseudotime of the NS keratinocytes.

d. Heatmap showing the five expression patterns of variable genes along the pseudotime of the PP keratinocytes.

e. Scatter plot showing the correlation between NS keratinocyte or PP keratinocyte pseudotimes and module scores calculated using cytokine target genes from the five expression patterns.

f. Scatter plot showing the correlation between PP keratinocyte pseudotimes and module scores calculated using genes induced in cultured keratinocytes stimulated by individual cytokines. The color represents the subtype identity of the cell.

Supplementary Fig. 7

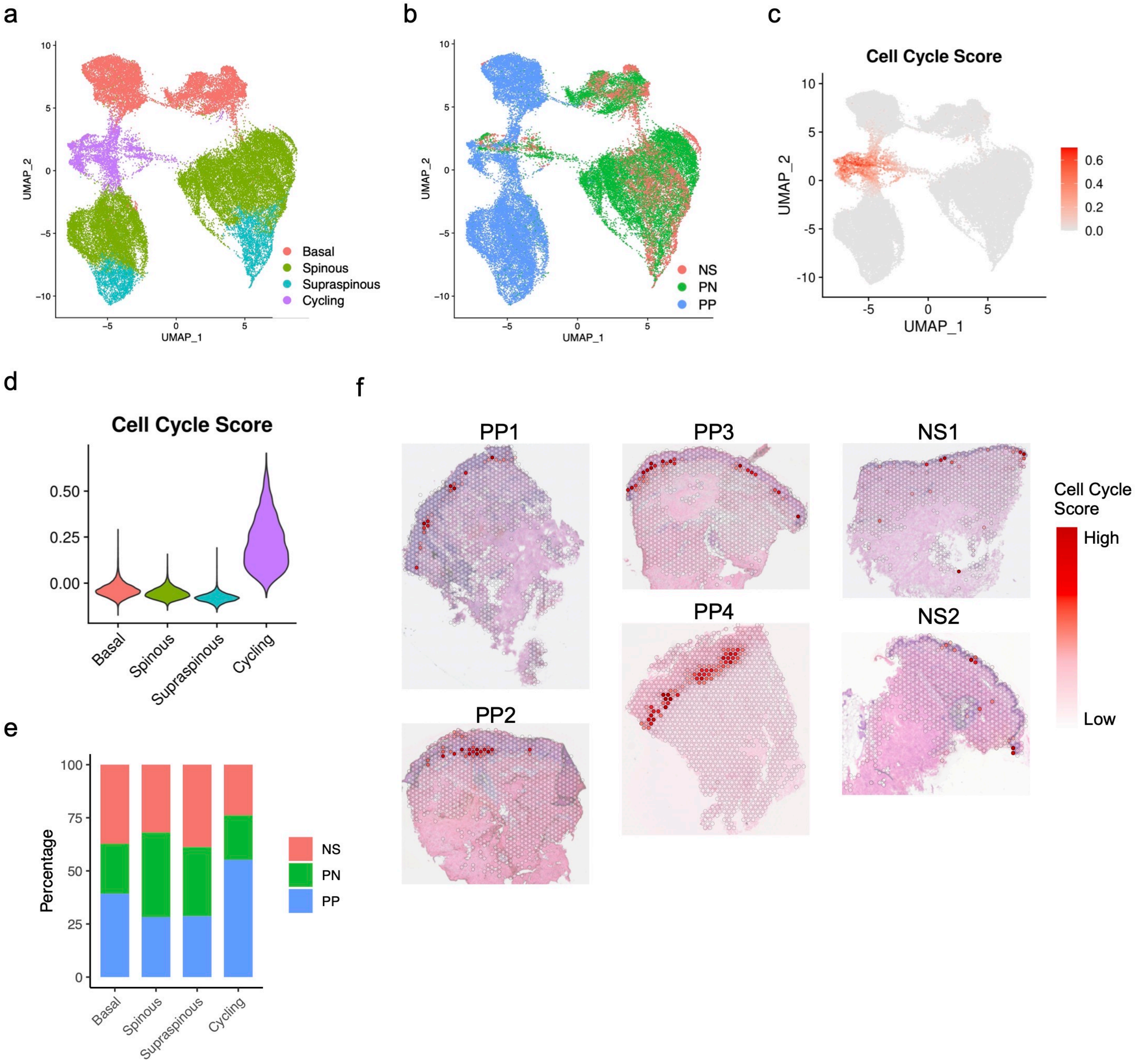

Supplementary Fig. 7. Keratinocyte cell cycle effects under the three disease conditions.

a. UMAP plot showing keratinocytes colored by cell types, the cycling keratinocytes were added to the previously annotated subtypes.

b. UMAP plot showing the keratinocytes colored by disease conditions.

c. UMAP plot showing the cell cycle score in the keratinocytes.

d. Violin plot showing the cell cycle score in the keratinocyte subtypes.

e. Bar plot showing the abundance composition across the disease conditions for each keratinocyte subtype.

f. Spatial plot showing the cell cycle module score in all the spatial-seq samples.

Supplementary Fig. 8

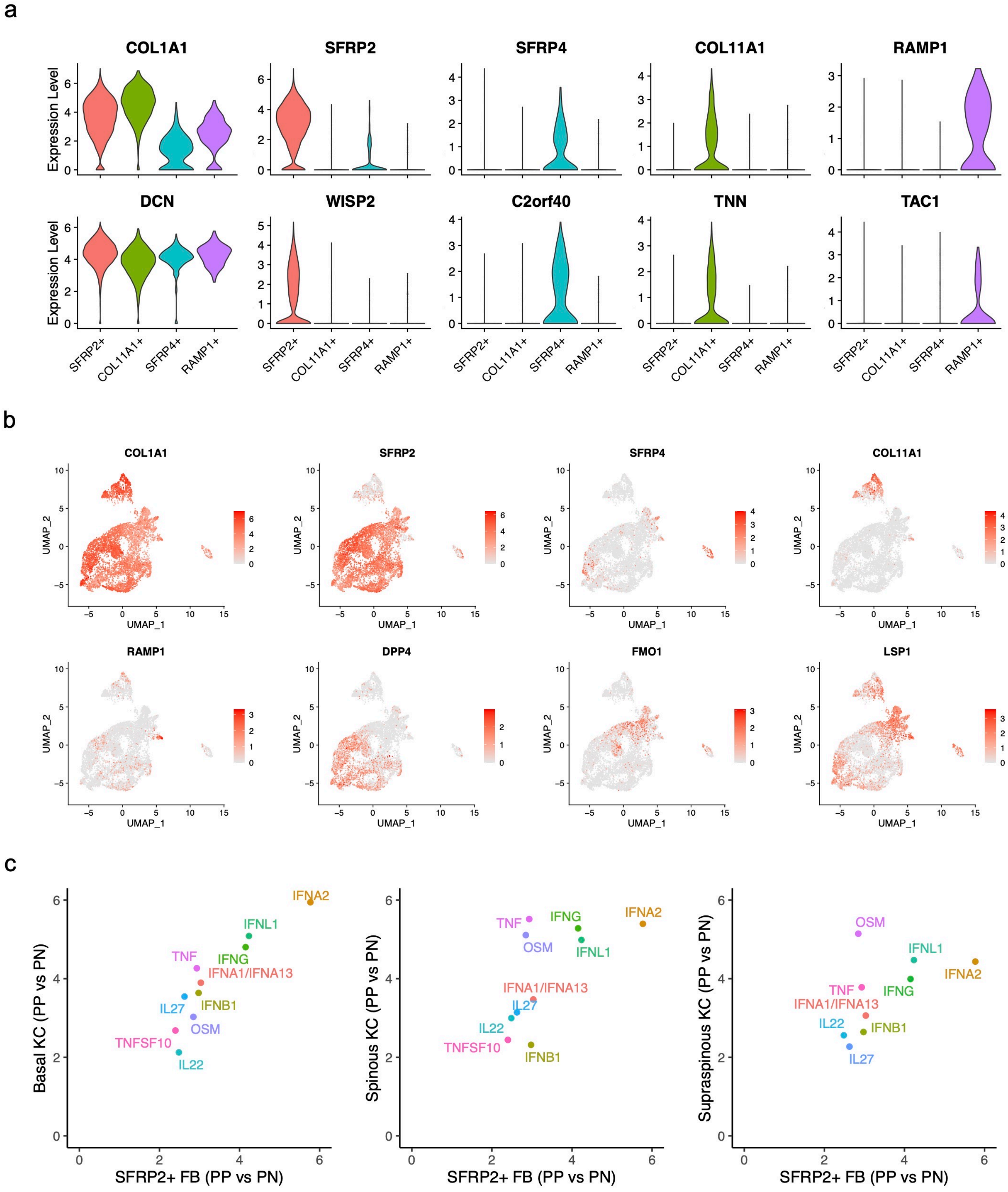

Supplementary Fig. 8. Identification of fibroblast subtypes.

a. Violin plots showing the expression level of representative marker genes for each fibroblast subtype.

b. UMAP showing the expression levels of representative marker genes in the fibroblast sub-clusters.

c. Scatter plots showing the activation z scores of common activated cytokine upstream regulators obtained by comparing PP to PN cells in the *SFRP2*$^{+}$ fibroblasts, basal keratinocytes, spinous keratinocytes, and supraspinous keratinocytes.

Supplementary Fig. 9

a

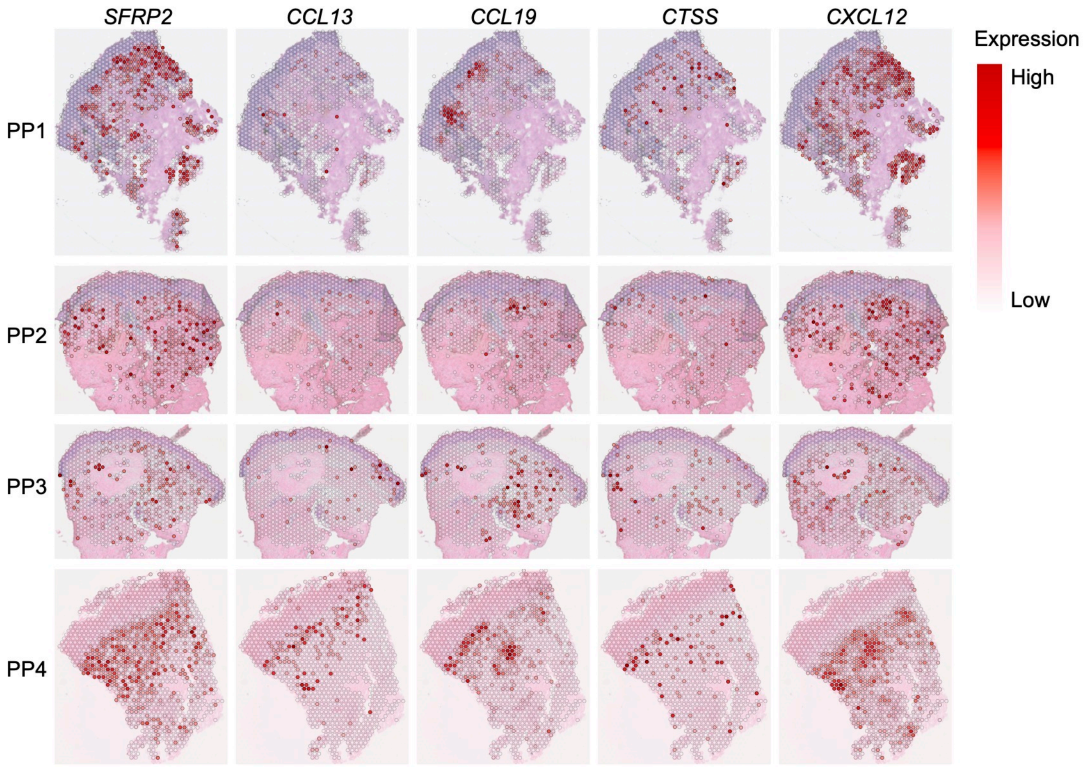

b

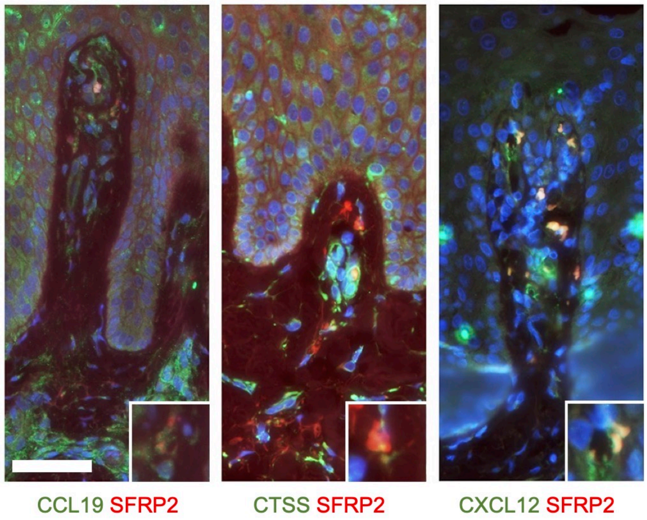

Supplementary Fig. 9. Spatial location of the fibroblast sub-clusters.

a. Spatial plots showing expression level of *SFPR2*, *CCL13*, *CCL19*, *CTSS*, and *CXCL12* in the PP spatial-seq samples.

b. Immunofluorescence showing the colocalization of CCL19, CTSS, and CXCL12 with SFRP2 in the dermal papillae.

Supplementary Fig. 10

a

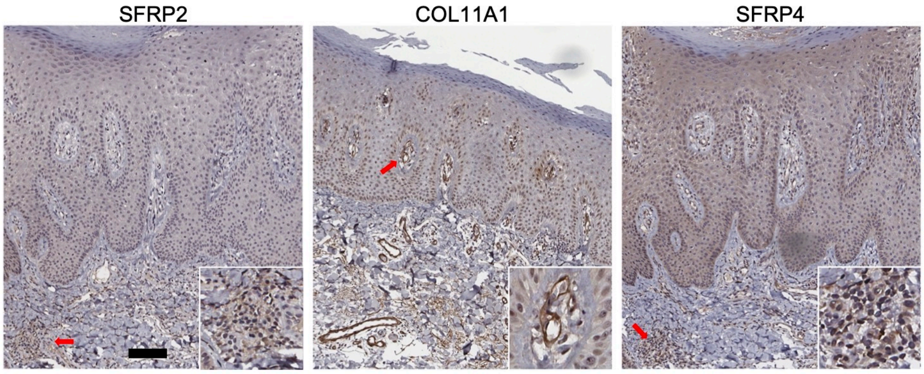

b

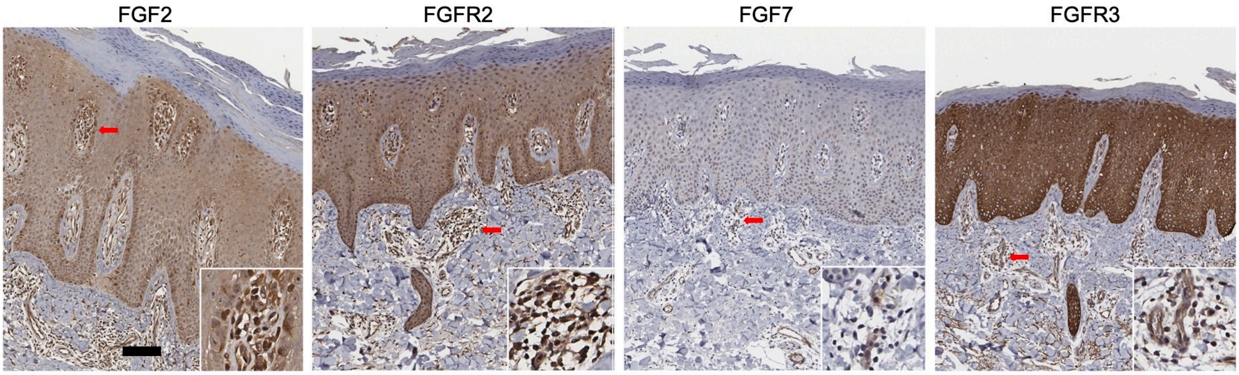

Supplementary Fig. 10. Immunohistochemistry for the fibroblast subtype markers, growth factor ligands and receptors.

a. Immunohistochemistry plots showing SFRP2, COL11A1 and SFRP4 in PP samples. The red arrow points to the zoom-in location. The size bar represents 100 μm.

b. Immunohistochemistry plots showing FGF2, FGF7, FGFR2 and FGFR3 in PP samples. The red arrow points to the zoom-in location. The size bar represents 100 μm.

Supplementary Fig. 11

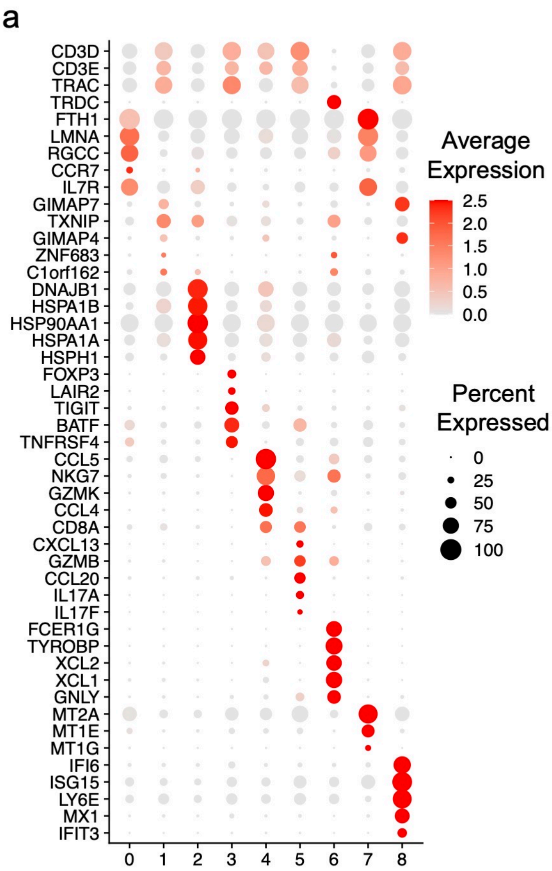

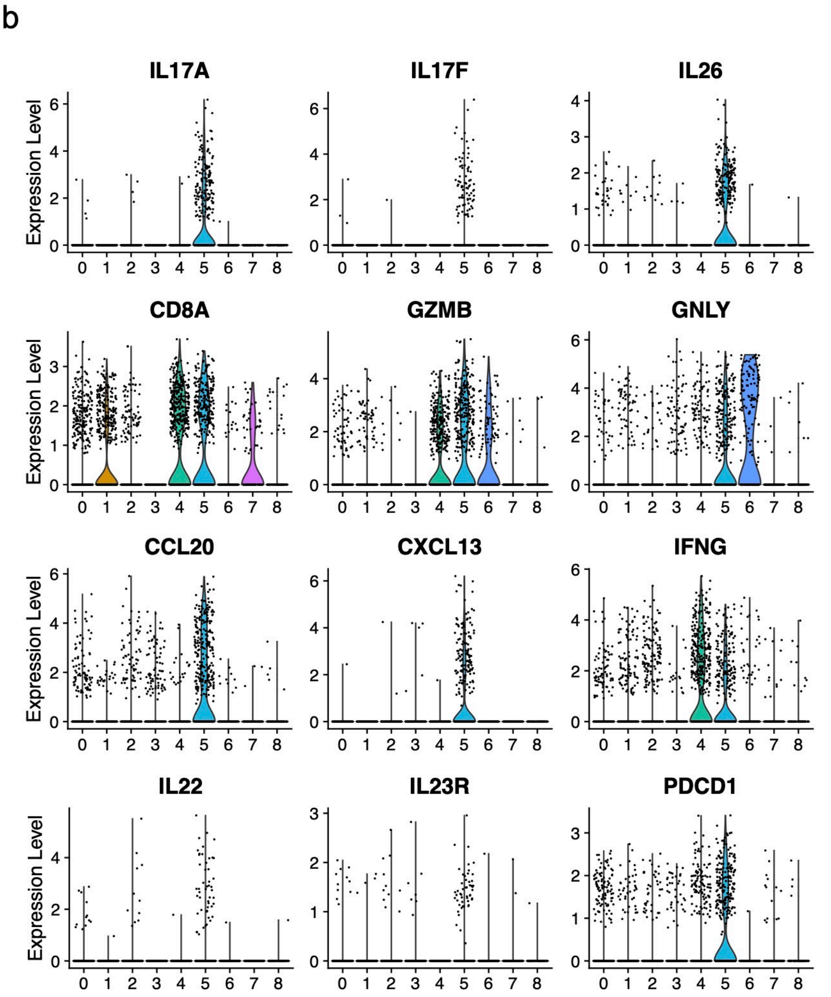

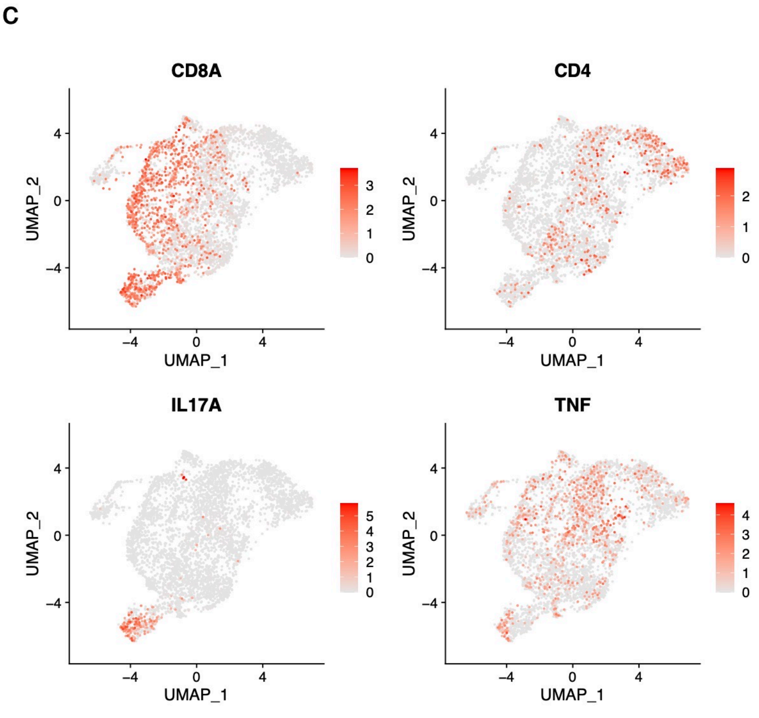

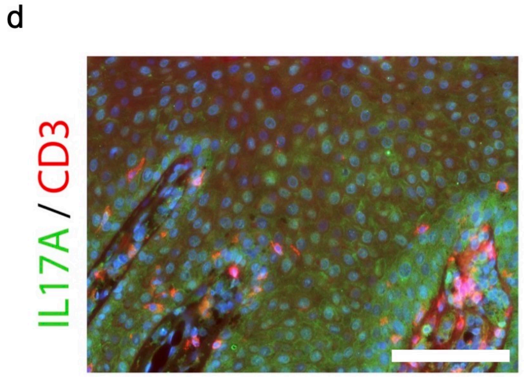

Supplementary Fig. 11. Identification of T cell subtypes by scRNA-seq and spatial-seq.

a. Dot plot showing the top marker genes for each sub-cluster. The color scale represents the scaled expression of gene expression. The size of the dot represents the percentage of T cells expressing the gene of interest.

b. Violin plot showing the expression of genes split by sub-cluster. Each dot represents the gene's expression in a single T cell.

c. UMAP plots showing the expression level of *CD8A*, *CD4*, *IL17A*, and *TNF* in the T cell subtypes.

d. Immunofluorescence showing the colocalization of CD3 with IL-17A in the dermal papillae.

Supplementary Fig. 12

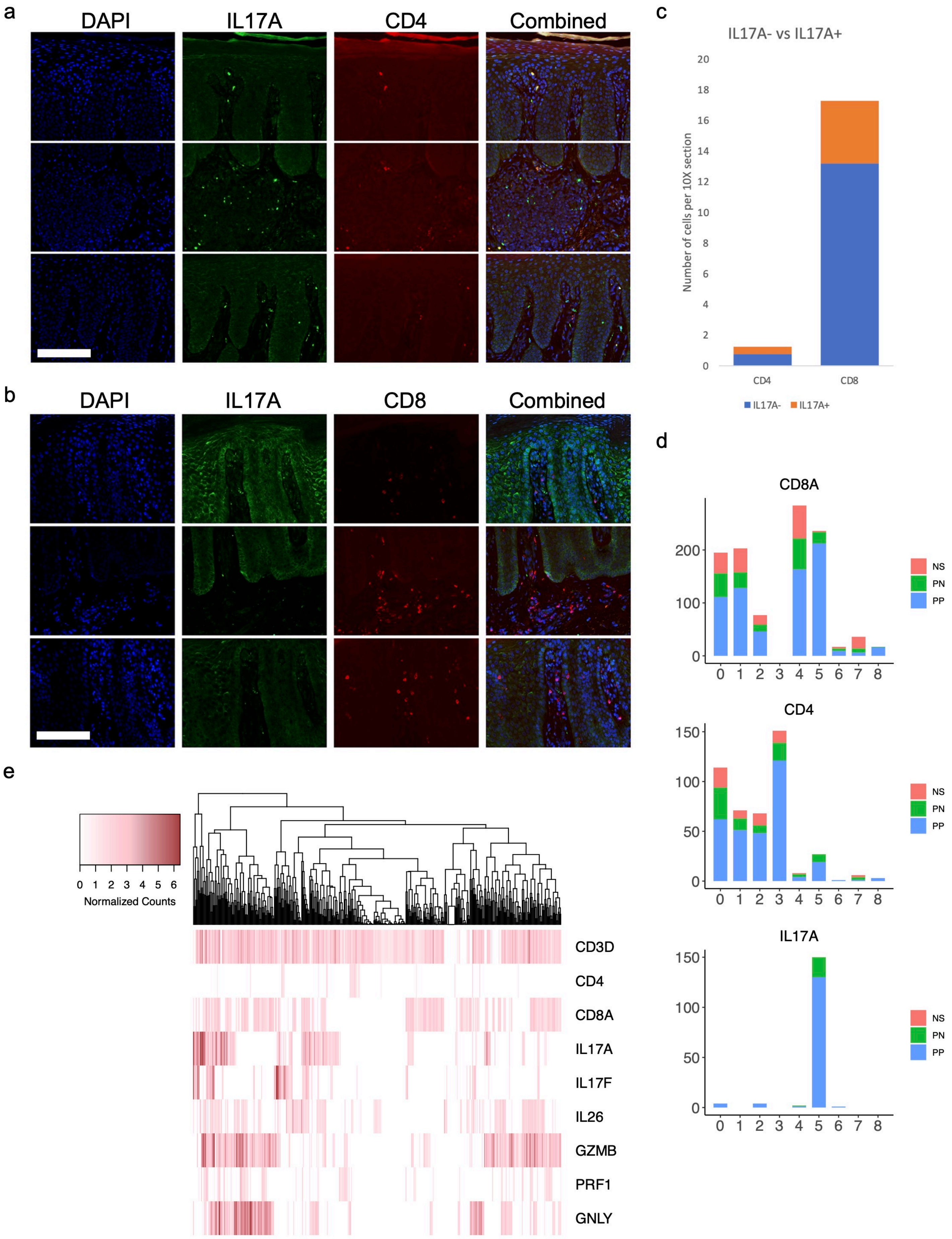

Supplementary Fig. 12. CD8+ Tc17 are a major source of IL17A in psoriatic skin.

a. Immunofluorescence plots showing IL17A and CD4 co-expression in PP samples.

b. Immunofluorescence plots showing IL17A and CD8 co-expression in PP samples.

c. Bar plot showing the number of cells in the immunofluorescence sections in a and b.

d. Bar plots showing the number of T cells expressing *CD8A*, *CD4*, and *IL17A* in the scRNA-seq data.

e. Heatmap showing the expression patterns of *CD3D*, *CD4*, *CD8A*, *IL17A*, *IL17F*, *IL26*, *GZMB*, *PRF1*, and *GNLY* across T cell sub-cluster 5.

Supplementary Fig. 13

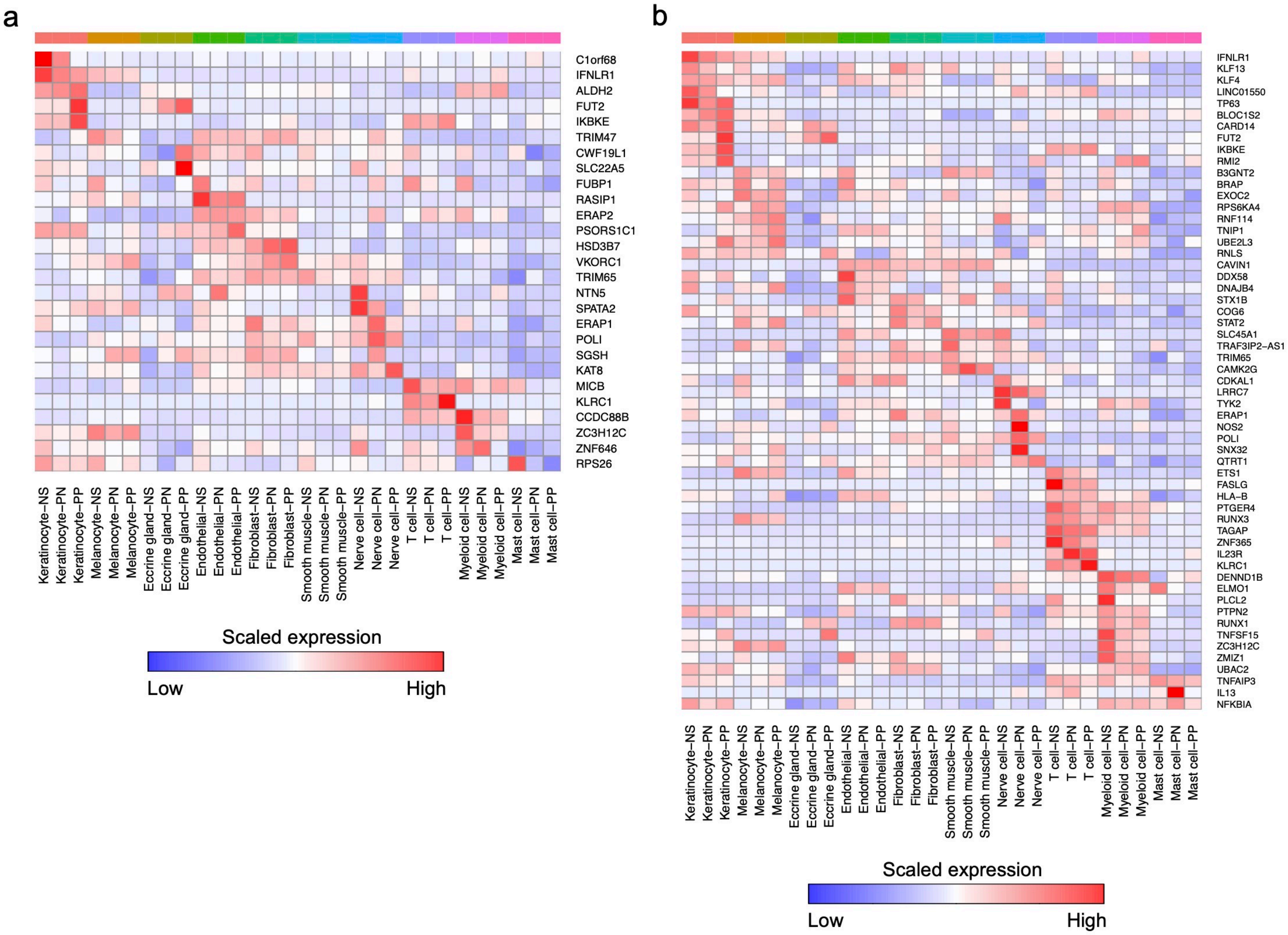

Supplementary Fig. 13. Cell type specificity of psoriasis associated genetic risk variants.

a. Heatmap showing the average expression of 27 genes associated with the psoriasis SNPs identified by eQTL analysis.

b. Heatmap showing the average expression of 57 nearest genes to the psoriasis SNPs.

Supplementary Fig. 14

a

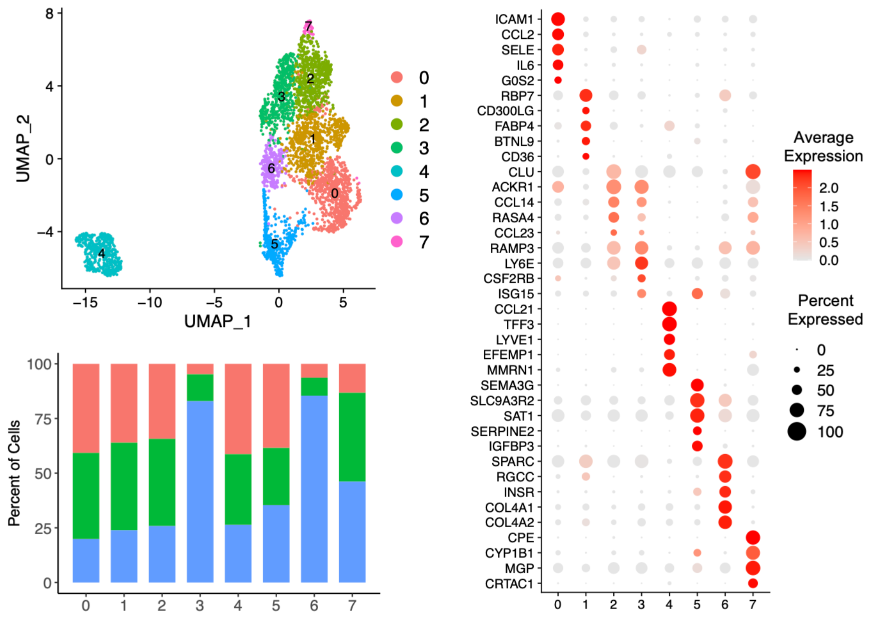

b

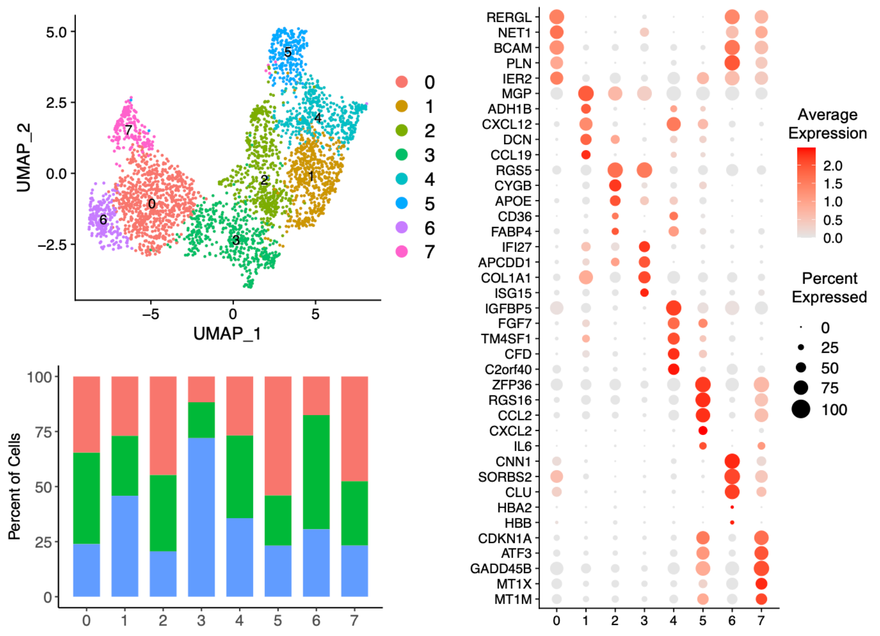

c

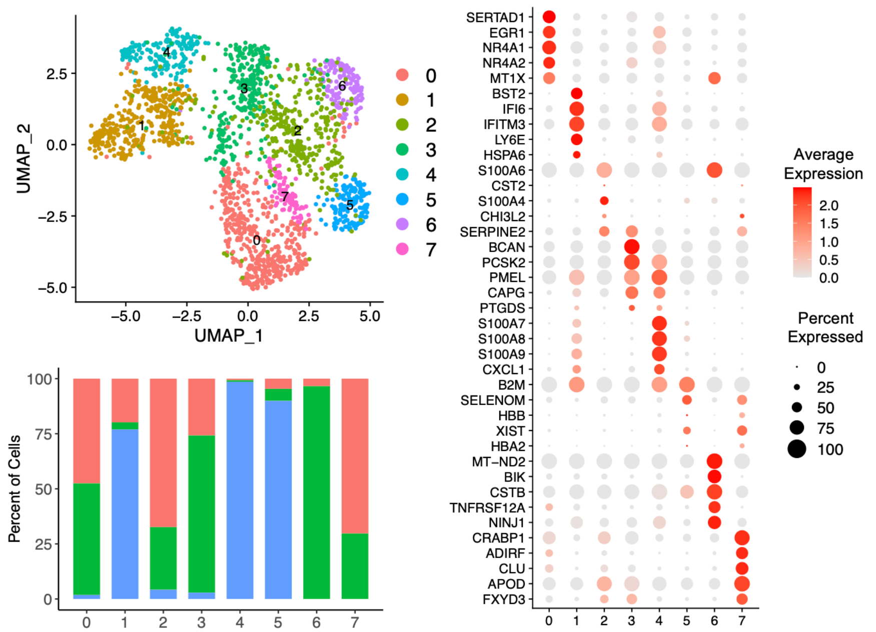

Supplementary Fig. 14. Identification of endothelial, smooth muscle and melanocyte subtypes.

a. UMAP plot showing 3,734 endothelial cells colored by sub-clusters. Bar plot showing the abundance composition across the disease conditions for each sub-cluster. Dot plot showing the top marker genes for each endothelial sub-cluster. The color scale represents the scaled expression of the gene. The size of the dot represents the percentage of endothelial cells expressing the gene.

b. UMAP plot showing 3,356 smooth muscle cells colored by sub-clusters. Bar plot showing the abundance composition across the disease conditions for each sub-cluster. Dot plot showing the top marker genes for each smooth muscle sub-cluster. The color scale represents the scaled expression of the gene. The size of the dot represents the percentage of smooth muscle cells expressing the gene of interest.

c. UMAP plot showing 1,987 melanocytes colored by sub-clusters. Bar plot showing the abundance composition across the disease conditions for each sub-cluster. Dot plot showing the top marker genes for each melanocyte sub-cluster. The color scale represents the scaled expression of the gene. The size of the dot represents the percentage of melanocytes expressing the gene.

Supplementary Fig. 15

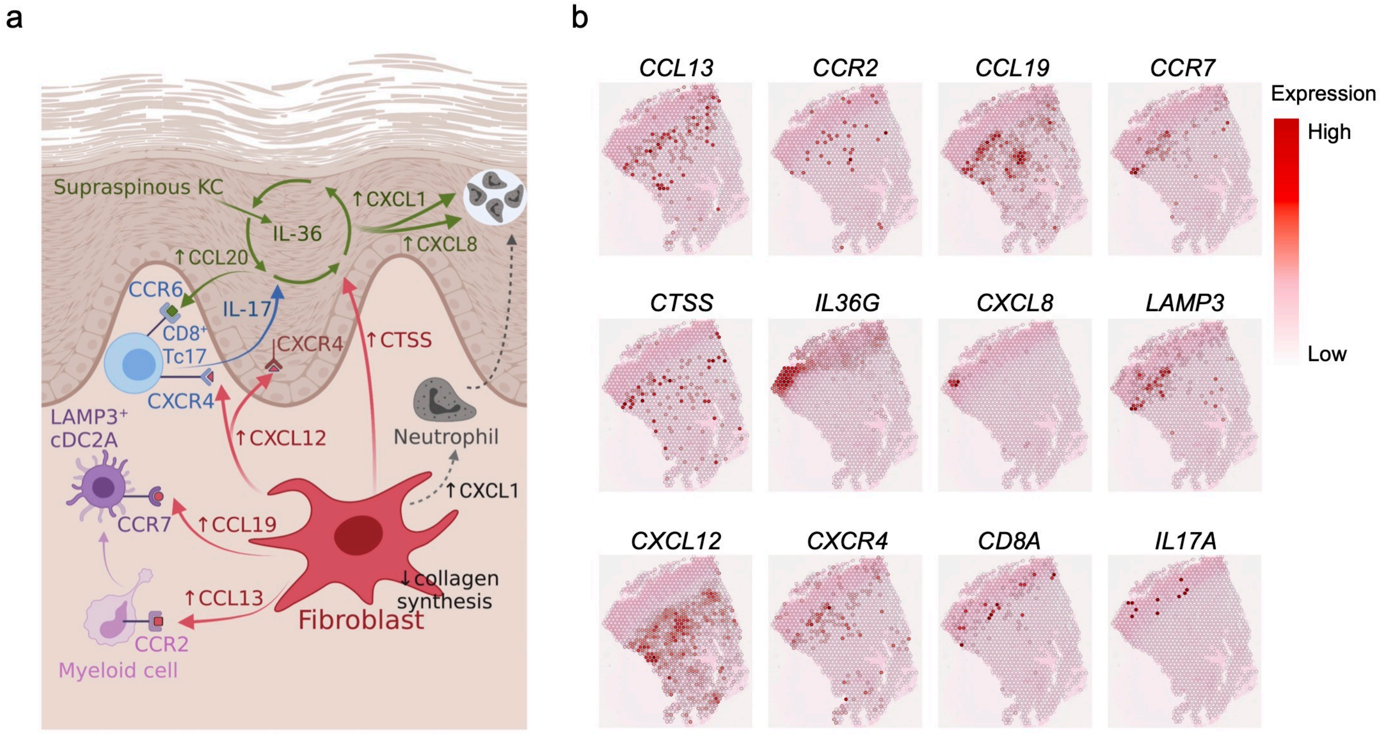

Supplementary Fig. 15. Schematic overview of the critical role of fibroblasts and the epidermis in promoting inflammatory responses in psoriatic skin.

a. Diagram linking the fibroblasts with the cell types involved in the inflammatory responses in the epidermis of the psoriatic skin. Neutrophils were linked with dotted lines because it was not detected in the scRNA-seq data but inferred based on the ligand-receptor analysis. Panel was created with BioRender.com.

b. Spatial plots showing expression level of cell type markers, key cytokines, and receptors in the interactions illustrated in a.
