# Supplementary material for: Single cell and spatial sequencing define processes by which keratinocytes and fibroblasts amplify inflammatory responses in psoriasis
Source: Nat Commun. 2023 Jun 12;14:3455. doi: 10.1038/s41467-023-39020-4 (PMC10261041; doi:10.1038/s41467-023-39020-4)
Supplement: Supplementary file 3 — Description of Additional Supplementary Files [file 41467_2023_39020_MOESM3_ESM.pdf]

Description of Additional Supplementary Files

Supplementary Data 1. Psoriasis sample demographics.

Supplementary Data 2. Quality metrics for the scRNA-seq, spatial-seq, and Seq-Scope datasets.

Supplementary Data 3. Cell type marker genes and the DEGs between NS and PP within each cell type for the scRNA-seq dataset. Wilcoxon rank sum test (two-sided) was used for the DEG analysis, and the Benjamini-Hochberg Procedure was used for false discovery rate adjustment.

Supplementary Data 4. Cluster marker genes for the six PP and NS spatial-seq datasets. Wilcoxon rank sum test (two-sided) was used for the DEG analysis, and the Benjamini-Hochberg Procedure was used for false discovery rate adjustment.

Supplementary Data 5. Keratinocyte subtype marker genes for the scRNA-seq dataset. Wilcoxon rank sum test (two-sided) was used for the DEG analysis, and the Benjamini-Hochberg Procedure was used for false discovery rate adjustment.

Supplementary Data 6. Differentially expressed genes between NS and PP in basal, spinous, and supraspinous keratinocytes in the scRNA-seq dataset. Wilcoxon rank sum test (two-sided) was used for the DEG analysis, and the Benjamini-Hochberg Procedure was used for false discovery rate adjustment.

Supplementary Data 7. Genes up-regulated by inducing keratinocytes with individual cytokines in bulk RNA-seq experiments.

Supplementary Data 8. Ligand-receptor pairs that had higher interaction scores in PP compared to NS among the keratinocyte subtypes in the scRNA-seq analyses.

Supplementary Data 9. Differentially expressed genes between NS keratinocytes and PP keratinocytes in the scRNA-seq and spatial-seq analyses. Wilcoxon rank sum test (two-sided) was used for the DEG analysis, and the Benjamini-Hochberg Procedure was used for false discovery rate adjustment.

Supplementary Data 10. Genes in five expression patterns along NS and PP keratinocyte pseudotimes in the scRNA-seq analyses.

Supplementary Data 11. Fibroblast subtype marker genes for the scRNA-seq dataset. Wilcoxon rank sum test (two-sided) was used for the DEG analysis, and the Benjamini-Hochberg Procedure was used for false discovery rate adjustment.

Supplementary Data 12. Differentially expressed genes between PN and PP cells in *SFRP2*$^{+}$ fibroblasts and the three keratinocyte subtypes in the scRNA-seq analyses. Wilcoxon rank sum test (two-sided) was used for the DEG analysis, and the Benjamini-Hochberg Procedure was used for false discovery rate adjustment.

Supplementary Data 13. Sub-cluster marker genes within PP fibroblast sub-cluster 0, 2, 3 and 6 in the scRNA-seq analyses. Wilcoxon rank sum test (two-sided) was used for the DEG analysis, and the Benjamini-Hochberg Procedure was used for false discovery rate adjustment.

Supplementary Data 14. T cell subtype marker genes for the scRNA-seq dataset. Wilcoxon rank sum test (two-sided) was used for the DEG analysis, and the Benjamini-Hochberg Procedure was used for false discovery rate adjustment.

Supplementary Data 15. Myeloid subtype marker genes for the scRNA-seq dataset. Wilcoxon rank sum test (two-sided) was used for the DEG analysis, and the Benjamini-Hochberg Procedure was used for false discovery rate adjustment.

Supplementary Data 16. Sub-cluster marker genes for endothelial cells, smooth muscle cells and melanocytes in the scRNA-seq analyses. Wilcoxon rank sum test (two-sided) was used for the DEG analysis, and the Benjamini-Hochberg Procedure was used for false discovery rate adjustment.

Supplementary Data 17. Ligand-receptor pairs that had higher interaction scores in PP compared to NS among the cell types in the scRNA-seq analyses.
