# Supplementary material for: Single cell and spatial sequencing define processes by which keratinocytes and fibroblasts amplify inflammatory responses in psoriasis
Source: Nat Commun. 2023 Jun 12;14:3455. doi: 10.1038/s41467-023-39020-4 (PMC10261041; doi:10.1038/s41467-023-39020-4)
Supplement: Supplementary file 5 — Reporting Summary [file 41467_2023_39020_MOESM5_ESM.pdf]

nature portfolio

Corresponding author(s): Johann E. Gudjonsson

Last updated by author(s): Apr 17, 2023

Reporting Summary

Nature Portfolio wishes to improve the reproducibility of the work that we publish. This form provides structure for consistency and transparency in reporting. For further information on Nature Portfolio policies, see our Editorial Policies and the Editorial Policy Checklist.

Statistics

For all statistical analyses, confirm that the following items are present in the figure legend, table legend, main text, or Methods section.

n/a	Confirmed	
☐	☒	The exact sample size (*n*) for each experimental group/condition, given as a discrete number and unit of measurement
☐	☒	A statement on whether measurements were taken from distinct samples or whether the same sample was measured repeatedly
☐	☒	The statistical test(s) used AND whether they are one- or two-sided<br>*Only common tests should be described solely by name; describe more complex techniques in the Methods section.*
☐	☒	A description of all covariates tested
☐	☒	A description of any assumptions or corrections, such as tests of normality and adjustment for multiple comparisons
☐	☒	A full description of the statistical parameters including central tendency (e.g. means) or other basic estimates (e.g. regression coefficient) AND variation (e.g. standard deviation) or associated estimates of uncertainty (e.g. confidence intervals)
☐	☒	For null hypothesis testing, the test statistic (e.g. *F*, *t*, *r*) with confidence intervals, effect sizes, degrees of freedom and *P* value noted<br>*Give P values as exact values whenever suitable.*
☒	☐	For Bayesian analysis, information on the choice of priors and Markov chain Monte Carlo settings
☐	☒	For hierarchical and complex designs, identification of the appropriate level for tests and full reporting of outcomes
☒	☐	Estimates of effect sizes (e.g. Cohen's *d*, Pearson's *r*), indicating how they were calculated

Our web collection on statistics for biologists contains articles on many of the points above.

Software and code

Policy information about availability of computer code

Data collection	No software was used for single cell or spatial-seq data acquisition.
Data analysis	10X Genomics software cellranger (version 3.1.0), spaceranger (version 1.3.0), R package Seurat (version 3.1.2), monocle (version 2.10.1), and python package CellphoneDB (version 2.0.0) were used to analyze the scRNA-seq and spatial-seq data. The functions from these packages and the parameters are reported in the methods section of the manuscript.

For manuscripts utilizing custom algorithms or software that are central to the research but not yet described in published literature, software must be made available to editors and reviewers. We strongly encourage code deposition in a community repository (e.g. GitHub). See the Nature Portfolio guidelines for submitting code & software for further information.

Data

Policy information about availability of data

All manuscripts must include a data availability statement. This statement should provide the following information, where applicable:

- Accession codes, unique identifiers, or web links for publicly available datasets
- A description of any restrictions on data availability
- For clinical datasets or third party data, please ensure that the statement adheres to our policy

The scRNA-seq data are available in GEO under accession number GSE173706. The Spatial-seq data are available in GEO under accession number GSE225475.

Field-specific reporting

Please select the one below that is the best fit for your research. If you are not sure, read the appropriate sections before making your selection.

[x] Life sciences   [ ] Behavioural & social sciences   [ ] Ecological, evolutionary & environmental sciences

For a reference copy of the document with all sections, see nature.com/documents/nr-reporting-summary-flat.pdf

Life sciences study design

All studies must disclose on these points even when the disclosure is negative.

Sample size	No sample size calculation was performed. The sample size for scRNA-seq and spatial-seq studies was determined by the availability of skin biopsies from psoriasis patient donors and healthy donors.
Data exclusions	Cells with less than 500 transcripts or 100 genes, or more than 10% of mitochondrial expression were filtered out as low-quality cells in scRNA-seq data analysis. After initial clustering of the remaining cells, clusters showing low transcript number and high mitochondrial expression were excluded to avoid the analysis of dead cells or empty droplets with fragments.
Replication	The samples in the same disease condition can be considered biological replicates. The replications were successful as the biological replicates showed similar gene expression profiles. There are in total 8 healthy, 11 perilesional and 14 lesional samples in scRNA-seq. There are 2 healthy and 3 lesional samples in spatial-seq. For the in vitro (cell culture) validation, three biological replicates were performed.
Randomization	Not applicable, as no comparison of experimental groups is performed. All comparisons presented are performed on cell groups from different disease conditions or different cell types.
Blinding	Blinding is not be applicable in the context of scRNA-seq and spatial-seq due to the technical complexity and exploratory nature of the techniques.

Reporting for specific materials, systems and methods

We require information from authors about some types of materials, experimental systems and methods used in many studies. Here, indicate whether each material, system or method listed is relevant to your study. If you are not sure if a list item applies to your research, read the appropriate section before selecting a response.

Materials & experimental systems

n/a	Involved in the study	
[ ]	[x]	Antibodies
[ ]	[x]	Eukaryotic cell lines
[x]	[ ]	Palaeontology and archaeology
[x]	[ ]	Animals and other organisms
[ ]	[x]	Human research participants
[x]	[ ]	Clinical data
[x]	[ ]	Dual use research of concern

Methods

n/a	Involved in the study	
[x]	[ ]	ChIP-seq
[x]	[ ]	Flow cytometry
[x]	[ ]	MRI-based neuroimaging

Antibodies

Antibodies used	Antibodies used include: CLEC9A (ThermoFisher Scientific, 55451-I-AP)(1:70), CLEC10A (ThermoFisher Scientific, TA810180) (1:150) (Clone:OTI2B10) , CD16 (Abcam, AB183354SP175)(2ug/ml)(Clone: SP175), CD163 (ThermoFisher Scientific, MA5-11458)(1:25) (Clone:10D6) , SFRP2 (Lifespan Biosciences, LS-C794043)(1:150) (Clone: OTI6E1), SFRP4 (Lifespan Biosciences, LS-C408100)(1ug/ml) (Epitope: aa22-303), COL11A1 (ThermoFisher Scientific, PA5-68410)(2ug/ml), Langerin (ThermoFisher Scientific, PA5-82422)(1:200), LAMP3 (ThermoFisher Scientific, PA5-84069)(1:50), FGFR3 (Abcam, AB231442)(2ug/ml), FGFR2 (ThermoFisher Scientific, 13042-I-AP) (2ug/ml), FGF2 (ThermoFisher Scientific, OSG00014W)(1:300), FGF7 (ThermoFisher Scientific, PA5-83670)(2ug/ml). IL-17A (Lifespan Biosciences, LS-C104427)(5ug/ml), CD4 (LifeSpan Biosciences, LS-C87801)(1:20) (Clone: 4B12), CD8 (Thermofisher scientific, MA5-13473)(1:50)(Clone: C8/144B), Goat IgG (Jackson ImmunoResearch Labs, 005-000-003), mouse IgG1 k (BioLegend, 400102) (Clone: MOPC-21)
Validation	Antibodies validated by the manufacturer were used. Antibodies were initially characterized and titrated by staining an appropriate positive control at several dilutions and a negative (isotype) control. Each image shown in the manuscript is representative of at least three biological replicates.

Eukaryotic cell lines

Policy information about cell lines

Cell line source(s)	N/TERT-2G, an immortalized keratinocytes cell line, was used with permission from James G. Rheinwald, Department of Dermatology, Brigham and Women's Hospital, Harvard Skin Disease Research Center, Boston, MA, United States.
Authentication	The N/TERT-2G line was developed in 2000 (see PMID:10648628), is widely published, and has been validated to express key epithelial differentiation markers. Beyond that no further authentication of this line has been performed.
Mycoplasma contamination	N/TERT-2G tested negative for Mycoplasma contamination.
Commonly misidentified lines (See ICLAC register)	This is a keratinocyte cell line, and there are no misidentified lines

Human research participants

Policy information about studies involving human research participants

Population characteristics	Skin biopsy specimens were obtained from patients with psoriasis at University of Michigan-Ann Arbor. The population characteristics are saved in supplementary table 1. Age matched healthy samples were collected for comparison.
Recruitment	Patients with psoriasis are seen at the hospital of University of Michigan-Ann Arbor. Patients were off systemic treatment and off any topical agents for at least 2 weeks prior to study time. Age matched healthy samples were collected for comparison. All patients/donors were asked if they wish to participate in the ongoing research studies, and all of them consented. No self-selection bias was presented in this study.
Ethics oversight	Informed written consent was obtained from human subjects under a protocol approved by the institutional review boards of University of Michigan-Ann Arbor. This study was conducted according to the Declaration of Helsinki Principles.

Note that full information on the approval of the study protocol must also be provided in the manuscript.
