# Supplementary material for: Single cell and spatial sequencing define processes by which keratinocytes and fibroblasts amplify inflammatory responses in psoriasis
Source: Nat Commun. 2023 Jun 12;14:3455. doi: 10.1038/s41467-023-39020-4 (PMC10261041; doi:10.1038/s41467-023-39020-4)
Supplement: Supplementary file 6 — Source Data [file 41467_2023_39020_MOESM6_ESM.zip › Source_Data/Supplementary_Data_Legends.docx]

**Source Data**

**Source Data 1. mRNA Expression values in Figure 2f.**

**Source Data 2. Original western blots, related to Supplementary Fig. 3g.**
